# Design Guidelines of a Computer-Based Intervention for Computer Vision Syndrome: Focus Group Study and Real-World Deployment

**DOI:** 10.2196/22099

**Published:** 2021-03-29

**Authors:** Youjin Hwang, Donghoon Shin, Jinsu Eun, Bongwon Suh, Joonhwan Lee

**Affiliations:** 1 Human Computer Interaction and Design Lab Seoul National University Seoul Republic of Korea; 2 Seoul National University Seoul Republic of Korea

**Keywords:** computer-based intervention, computer vision syndrome, system interface, deployment study

## Abstract

**Background:**

Prolonged time of computer use increases the prevalence of ocular problems, including eye strain, tired eyes, irritation, redness, blurred vision, and double vision, which are collectively referred to as computer vision syndrome (CVS). Approximately 70% of computer users have vision-related problems. For these reasons, properly designed interventions for users with CVS are required. To design an effective screen intervention for preventing or improving CVS, we must understand the effective interfaces of computer-based interventions.

**Objective:**

In this study, we aimed to explore the interface elements of computer-based interventions for CVS to set design guidelines based on the pros and cons of each interface element.

**Methods:**

We conducted an iterative user study to achieve our research objective. First, we conducted a workshop to evaluate the overall interface elements that were included in previous systems for CVS (n=7). Through the workshop, participants evaluated existing interface elements. Based on the evaluation results, we eliminated the elements that negatively affect intervention outcomes. Second, we designed our prototype system LiquidEye that includes multiple interface options (n=11). Interface options included interface elements that were positively evaluated in the workshop study. Lastly, we deployed LiquidEye in the real world to see how the included elements affected the intervention outcomes. Participants used LiquidEye for 14 days, and during this period, we collected participants’ daily logs (n=680). Additionally, we conducted prestudy and poststudy surveys, and poststudy interviews to explore how each interface element affects participation in the system.

**Results:**

User data logs collected from the 14 days of deployment were analyzed with multiple regression analysis to explore the interface elements affecting user participation in the intervention (LiquidEye). Statistically significant elements were the instruction page of the eye resting strategy (*P*=.01), goal setting of the resting period (*P*=.009), compliment feedback after completing resting (*P*<.001), a mid-size popup window (*P*=.02), and CVS symptom-like effects (*P*=.004).

**Conclusions:**

Based on the study results, we suggested design implications to consider when designing computer-based interventions for CVS. The sophisticated design of the customization interface can make it possible for users to use the system more interactively, which can result in higher engagement in managing eye conditions. There are important technical challenges that still need to be addressed, but given the fact that this study was able to clarify the various factors related to computer-based interventions, the findings are expected to contribute greatly to the research of various computer-based intervention designs in the future.

## Introduction

### Background

As computer technologies advance rapidly, an increasing number of people spend their time in front of computer screens and mobile phones. According to the current population survey, 89% of US households have a computer, which includes smartphones, and 81% have a broadband internet subscription [1]. There is also computer use increase in the workplace. It is estimated that more than 75% of all jobs involve computer use [2]. Before personal computers revolutionized the workplace, office work had involved a range of activities, including typing, filing, reading, and writing. Each activity was adequately varied in the requirements of posture and vision, posing a natural “break” from the previous activity.

However, the introduction of personal computers has combined these tasks to where most can be performed without moving from the desktop, thereby improving quality, production, and efficiency, but also increasing computer-related health issues [2].

Computer vision syndrome (CVS) is one of the typical computer-related health issues [3-6]. Approximately 70% of computer users have CVS-related problems. The American Optometric Association defines CVS as the combination of eye and vision problems associated with the use of computers. The ocular complaints made by computer users typically include eye strain, eye fatigue, burning sensation, irritation, redness, blurred vision, and dry eyes, among others. The condition of a person experiencing one or more of these ocular complaints as a result of operating a computer and looking at a computer monitor is generally referred to as CVS. Symptoms of CVS also include extraocular symptoms, such as neck pain, back pain, and shoulder pain [7,8]. All these symptoms negatively affect the performance of everyday tasks, such as reading, driving, and computer use, which lowers quality of life [9].

Designing an appropriate intervention involving eye rest is one of the technology-based solutions to reduce the prevalence of CVS [10-13]. Among the various forms of interventions, a computer-based intervention is an appropriate form of intervention for CVS [9]. A computer-based intervention offers a great variety of options for assessing individuals, creating and delivering customized health messages, and providing individuals with the methods necessary to maintain or change their health-related behaviors [14]. Maximizing benefits and minimizing costs are important when designing health interventions, including digital health interventions such as computer-based interventions [15-19]. Inadequate design is one of the reasons for increasing costs in the process of modifying and re-evaluating interventions [19,20]. Therefore, a design study for effective computer-based interventions should be performed before introducing a prototype to users [21].

### Objectives

In this study, we aimed to explore interface elements that affect participation in a computer-based intervention helping the eye resting behavior of users with CVS. For this, first, we investigated effective interface elements in existing computer-based interventions for users with CVS during a focus group study. Second, to further investigate the effectiveness of interface elements, we conducted a deployment study with our prototype LiquidEye having multiple interface options. Interface options included interface elements that were evaluated higher than the average in the focus group study. To demonstrate how the included elements affect user participation in eye resting behavior, we deployed LiquidEye in the real world with 12 participants.

## Methods

### Study Procedure

This study included a focus group study and a deployment study. To research existing computer-based interventions for vision protection, screening was conducted before the focus group. In this phase, researchers screened and listed the interface elements from existing systems. In the focus group consisting of an evaluation session and a redesign session, participants with CVS (n=7) evaluated each element by discussing its pros and cons. The evaluation session was conducted with a focus group interview. In the redesign session, participants discussed additional interface elements that could affect system participation and all participants evaluated the elements. With interface elements rated higher than the average, we developed LiquidEye and conducted a deployment study with 12 participants.

### Screening Existing Systems

During the screening phase, researchers aimed to list feasible interface elements from existing systems. Our system selection criteria were designed through a four-step procedure. In step 1, we collected all previously studied systems regarding CVS in the human-computer interaction community or other related fields. There were systems such as EyeGuardian [22], EyePhone [23], DualBlink [9], LiDAR [5], BlinkBlink [24], and EyeProtector [25]. In step 2, apps from the app store or web-based interventions were collected, such as ProtectYourVision, RestOnTime, and EyeBreak. In step 3, systems with all the intervening effects were collected and preanalyzed by researchers. Finally, in step 4, three systems were selected to include as many elements as possible and minimize overlap between systems. Step 4 was conducted because showing too many systems in one place could confuse participants. In the end, three systems were included in the focus group session. One was a prior academic prototype (Eye Protector [25]) and two were obtained from a commercial app store (Protect Your Vision [26] and Rest on Time [27]).

### Phase 1: Focus Group Study

To evaluate and discuss interface elements in existing computer-based interventions for CVS, we conducted a focus group discussion. We recruited seven participants (three male and four female participants; P1-P7) aged from 21 to 37 years (Table 1). All participants reported that they had frequently experienced CVS symptoms, such as blurred vision, dry eyes, eye strain, headache, neck pain, and back pain [4,7]. Recruited participants experienced at least three of these symptoms. Additionally, they were using a computer for at least 3 hours a day. We recruited participants through the university’s online community and clinical recruiting sites. Each participant was given a US $30 voucher after completing the final session. The focus group discussion consisted of two major sessions (evaluation session and redesign session).

**Table 1:** Information of the participants in the focus group study.

Participant number	Age (years)	Gender	Average computer use per day	Related symptoms
P1	29	Female	≥4 h	Blurred vision, dry eyes, eye irritation, and neck and back pain
P2	28	Male	≥4 h	Blurred vision, dry eyes, headache, and neck and back pain
P3	24	Female	≥4 h	Blurred vision, double vision, dry eyes, eye irritation, headache, and neck and back pain
P4	22	Female	≥2 h	Blurred vision, dry eyes, eye irritation, headache, and neck and back pain
P5	26	Male	≥3 h	Blurred vision, dry eyes, eye irritation, and neck and back pain
P6	21	Female	≥4 h	Blurred vision, dry eyes, and neck and back pain
P7	37	Male	≥4 h	Blurred vision, double vision, and dry eyes

#### Evaluation Session

In this session, participants were asked to evaluate the interface elements in existing systems with other participants. We introduced the three intervention systems for CVS. For each system, the included interface elements and their functions were described to participants in detail with a simulation. Thereafter, participants discussed each interface element in detail and mainly discussed its acceptability, which is an important consideration for health technologies and interventions [28]. Acceptable interventions make users more likely to engage and adhere to the system. Participants evaluated the acceptability of each interface element on a 7-point Likert scale.

#### Redesign Session

The objective of the redesign session was to explore additional interface elements that were not included in previous systems. Participants were instructed to draw their ideal intervention system on a paper. They were told that they could take some of the factors they evaluated in the previous session or add new ones if needed. This enabled us to further discover and evaluate new important elements that could not be considered in the previous session. The participants drew what they thought was a desirable system on a given blank sheet of paper. In this session, participants were also instructed to focus on the acceptability of the system. To consider as many factors as possible, a researcher did not give participants a preannounced time and waited until all participants had finished their drawings. It took a total of 20 minutes. Thereafter, the participants explained their desirable system and interface elements to other participants and two of the authors (YJ and DH). Newly suggested elements were listed by the authors, and participants evaluated these elements as they did in the evaluation session.

### Phase 2: Deployment Study

#### LiquidEye: Computer-Based Intervention for Users With CVS

LiquidEye is a computer-based intervention system that helps achieve an adequate amount of eye rest among users with CVS. It helps users’ eye resting behavior by providing an intervening screen with a black/white full-screen window. The goal of LiquidEye is to minimize vision-related symptoms and prevent the occurrence of vision-related symptoms. For this, LiquidEye attempts to manage a user’s prolonged time of computer use, which is one of the critical causes of CVS [7].

**Figure 1:**
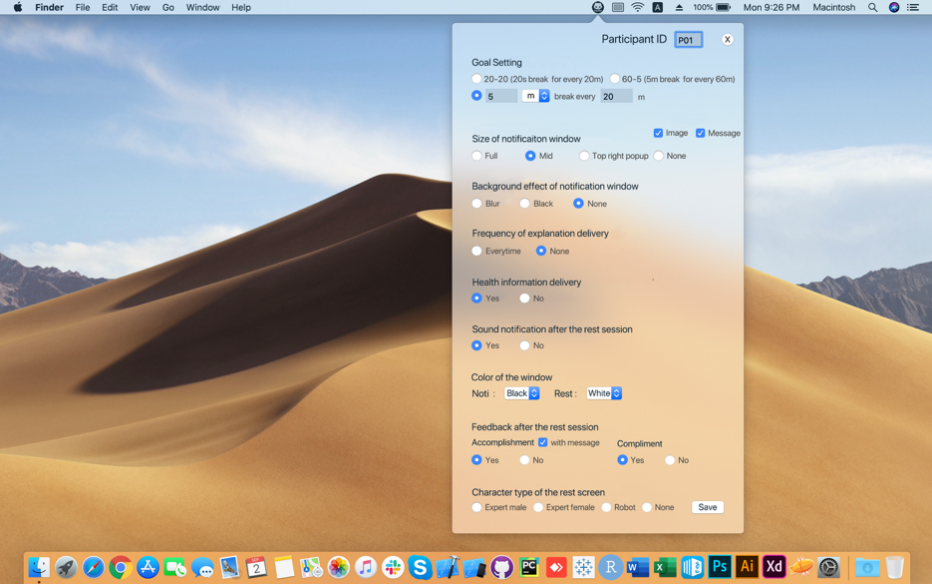
Settings menu of LiquidEye. In the settings menu, users can customize their interface options for LiquidEye.

LiquidEye consists of multiple interface options. Interface options include interface elements that were evaluated higher than the average in the focus group study. As shown in Figure 1, users can select these options on the settings menu or can adjust the degree of each interface element (frequency, size, etc). Based on the user’s settings, LiquidEye intervenes in prolonged computer use at the scheduled time with selected interface elements.

**Figure 2:**
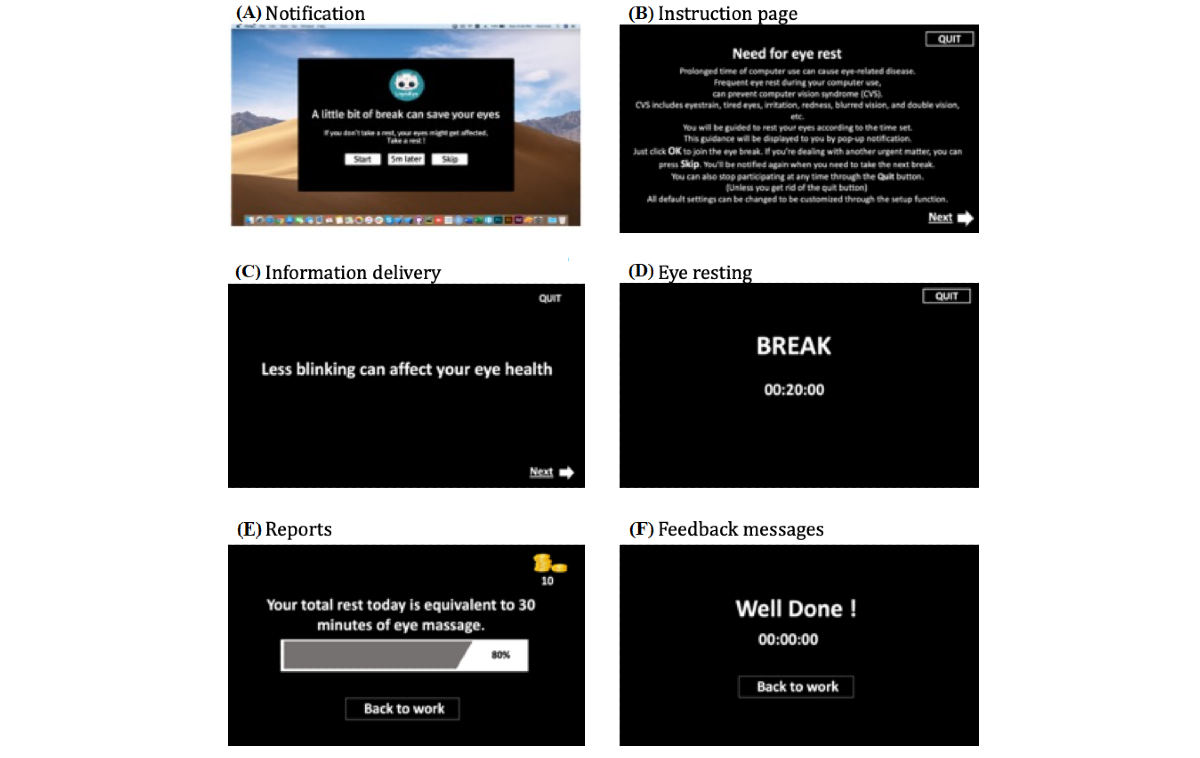
Example scenarios of LiquidEye. The interface elements (A-F) in the example scenarios are customizable in the settings menu.

Figure 2 shows example scenarios of LiquidEye. At the scheduled time, a notification interface pops up and asks the user to participate in eye rest (Figure 2A). Depending on the user’s customized settings, this element can accompany symptom-like visual effects. Users can choose among “start,” “5 min later,” and “skip.” If the user clicks “start,” LiquidEye records the user’s behavior as “1 (participated),” and if the user clicks “skip,” it records the user’s behavior as “0 (did not participate).” When the user clicks “5 min later,” the notification window interface pops up 5 minutes later. If the user turns off the notification window option, the eye resting scenario starts without a notification interface. Before starting eye rest, LiquidEye shows an instruction page that explains the need for eye rest and how to use LiquidEye (Figure 2B) and provides health information related to CVS (eg, less eye blinking can cause CVS, foods with beta-carotene can help improve eye conditions, etc) (Figure 2C). The instruction page and health information are optional depending on the user’s customized settings. During eye rest, the word “break” appears and the word “look away” appears next and remains on the screen (Figure 2D). There is a timer in the middle that shows the remaining time for the user’s eye resting behavior. Characters could be presented on the screen depending on the option settings. After the resting time, the user can receive a sound-based alarm (optional). If the user quits the LiquidEye window (button on the top right) before the eye resting time is over, it records the user’s behavior as “0 (did not participate).” If the user finishes eye resting without quitting, LiquidEye shows the user’s eye resting accomplishment report for the day (Figure 2E). A rotated feedback message (compliment) can be sent to the user depending on the settings (Figure 2F).

LiquidEye was implemented on the MacOS system, and the system was developed with Swift in Xcode 10.3 IDE. After the development of LiquidEye, we conducted a 14-day real-world study with 12 recruited participants.

#### Participants and Procedure

A total of 12 participants (seven male and five female participants; A1-A12) aged from 22 to 40 years with CVS were recruited (Table 2). They all had at least three CVS-related symptoms and were using the computer for at least 3 hours a day. Participants were recruited through an online community. Participants in the focus group study did not overlap with participants in the deployment study. LiquidEye was used by participants for 2 weeks. Before participants started to use LiquidEye, they visited our lab to participate in a prestudy interview. Additionally, we helped participants to download LiquidEye on their personal computers and check if LiquidEye works properly on the device. To make participants use as many elements as possible, we instructed participants to use the system by changing the setting environment at least twice a day for an everyday task. Moreover, each time they changed the settings, they were asked to enter feedback for the previously used interface elements on an automatically appearing feedback page. Participants also visited the authors after the 14 days of participation for a postuse interview.

**Table 2:** Information of the participants in the 14-day deployment study.

Participant number	Age (years)	Gender	Average computer use per day	Number of reported CVS^a^ symptoms	Occupation
A1	28	Female	≥4 h	4	Student
A2	31	Male	≥7 h	4	Data scientist
A3	22	Female	≥5 h	4	Student
A4	24	Female	≥3 h	3	Student
A5	23	Male	≥3 h	3	Office worker
A6	28	Female	≥5 h	3	Student
A7	28	Male	≥3 h	3	Data scientist
A8	24	Male	≥4 h	4	Student
A9	30	Male	≥7 h	3	Programmer
A10	33	Male	≥5 h	3	Office worker
A11	24	Female	≥4 h	3	Student
A12	40	Male	≥3 h	4	Office worker

^a^CVS: computer vision syndrome.

#### Thematic Analysis of Qualitative Data

Qualitative data that were collected through semistructured interviews and users’ real-time feedback during LiquidEye use were integrated and analyzed. With these data, we conducted a thematic analysis to increase understanding. To build up themes from the data, we referred to the seven-element constructs of the Theoretical Framework for Acceptability developed by Sekhon et al [28]. These constructs consist of (1) affective attitude (how an individual feels about the intervention), (2) burden (perceived amount of effort that is required to participate in the intervention), (3) ethicality (extent to which the intervention has a good fit with an individual’s value system), (4) intervention coherence (extent to which the participant understands the intervention and how it works), (5) opportunity cost (extent to which benefits, profits, or values must be given up to engage in the intervention), (6) perceived effectiveness (extent to which the intervention is perceived as likely to achieve its purpose), and (7) self-efficacy (participants’ confidence that they can perform the behaviors required to participate in the intervention).

#### Statistical Analysis of Participation Depending on Interface Elements

With the user data logs collected during LiquidEye use, we conducted quantitative analysis. To investigate if each interface element significantly affects user participation in eye resting behavior suggested by the LiquidEye system (1: participated, 0: did not participate), multiple regression analysis was conducted. Interface elements were analyzed as independent variables, and participation was analyzed as a dependent variable. We conducted statistical analyses using R software (R Foundation for Statistical Computing).

## Results

### Results From Phase 1 (Focus Group Study)

Table 3 shows the list of interface elements evaluated in the focus group study and their ratings, including newly suggested interface elements in the redesign session during the focus group discussion. We classified these elements into subthemes and themes. During the classification process, we referred to the behavioral intervention technology model that involves frameworks integrating the conceptual framework into the technological framework [29].

**Table 3:** List and scores of interface elements that resulted from the focus group study.

Theme	Subthemes	Interface elements (score)^a^	Related systems
Behavior change strategies	Education	Instruction (4.0)	Rest on Time
Behavior change strategies	Goal setting	Goal selection (5.7)	Protect Your Vision
Behavior change strategies	Monitoring	Participation (4.8)	Rest on Time
Behavior change strategies	Feedback	Descriptive message (4.0) Comparative message (+) (4.2) Evaluative message (+) (4.2) Compliment message (4.8)	Rest on Time Protect Your Vision
Behavior change strategies	Reward	Monetary reward (+) (5.0) Score reward (+) (4.2)	N/A^b^
Elements	Information delivery	Health information (+) (6.0)	N/A
Elements	Notification	Popup (4.8) Full screen (4.3)	Rest on Time
Characteristics	Medium	Physical signal (−) (2.2) Sound (4.0) Screen based (6.2)	Protect Your Vision Eye Protector Rest on Time
Characteristics	Complexity	Rotated message (+) (6.0)	N/A
Characteristics	Aesthetics	An agent with robot appearance (4.0) An agent with expert appearance (+) (5.2) Spot effect (−) (2.8) Flashing effect (−) (2.8) Blurred effect (4.8) Symptom-like effect (+) (5.7)	Protect Your Vision Eye Protector
Workflow	User defined	Customization (+) (6)	N/A
Workflow	Conditions	20-20-20 (4.8) 60-5 (4.0) Just-in-time (+) (4.2)	Protect Your Vision Eye Protector Rest on Time

^a^Interface elements with low effectiveness are marked with “−,” and interface elements newly added during the focus group discussion are marked with “+.”

^b^N/A: not applicable.

#### Interface Elements With Low Effectiveness

Elements with low effectiveness (score below 4.0) were not included in LiquidEye. Interface elements with low effectiveness were visual effects of spot and flashing, and physical signal popups at the scheduled resting time (marked with “−” in Table 3). The spot is a feature that involves a colored dot icon intended to minimize interruption depending on the user’s condition. Participant P6 made the following statement:

I don't think it's going to be noticeable. It only takes up a small part of the screen.

The flashing effect was also discussed as below effective for the same reason as the spot. For physical signals, such as blowing wind toward the user’s eyes, it was discussed as effective for grabbing the user’s attention, but most participants rated it with a low score owing to its annoying interruption.

#### Newly Added Interface Elements

Additional interface elements were discussed in the focus group discussion (marked as “+” in Table 3). Symptom-like effects were suggested by participant P6. The participant made the following statement:

If the visual effect in the screen-based intervention come up with the CVS symptom like effects such as blurred vision or black spot, it will increase susceptibility to CVS, thus increase participation.

The reward element was suggested by participant P2, participant P4, and participant P5. Participant P4 made the following statement:

Like playing the game, the rewards of making virtual money or getting high scores will affect not only early acceptability but also motivation for long-term use.

For health information, participant P1, participant P5, and participant P6 indicated the need for this element. Participant P5 made the following statement:

Medical center does not usually give detailed eye-resting instructions. If we can get health information through this system, we can eventually make more efforts to improve CVS related symptoms.

Participant P2 and participant P6 suggested a character with an expert-like appearance in the stage of eye resting instructions. Participant P6 made the following statement:

Expert-like character will increase the credibility of the information follows.

A just-in-time function was suggested from the paper prototype of participant P4. Participant P4 made the following statement:

It would be more acceptable if the system has the function of avoiding important time such as meeting time.

A customization option was suggested by most of the participants. Participant P1, participant P2, participant P4, participant P5, and participant P6 added customization options to their paper prototypes. Participant P6 made the following statement:

Different people have different demands for designs and functions, so it would be better if we could select the elements at the beginning of the system use.

Rotation of messages in the system was suggested by participant P1, participant P2, participant P3, participant P6, and participant P7. Participant P1 made the following statement:

Rotated messages will make the system more useful.

#### Additional Comments

After listing all interface elements in the focus group discussion, additional comments were collected to design LiquidEye. We conducted a focus group interview to discuss how the final elements (score above 4.0; to be implemented in LiquidEye) should be customized for the users. There existed several comments about varying frequency, varying interface size or design, and adding customization options. We present these results in line with the themes in Table 3.

For the education element (instruction on the system and how to use it), participants anticipated that the presence of this element matters more than how the element itself is organized. Some participants said they do not need it at all, while others said they want it to be for a specific period of time. Thus, two options, one with and one without the element, were implemented as customizable in LiquidEye.

For goal setting, which is setting resting frequency and the time of the day, most participants insisted that it should be customizable. In our case, reducing symptoms of CVS was our major clinical aim. To prevent CVS caused by prolonged computer use, clinical optometrists suggest users follow the 20/20/20 rule [30], which is that one should look at something 20 feet away for at least 20 seconds after 20 minutes of computer use [31]. However, since it is not easy to follow these guidelines, participants mentioned that they need flexibility with eye resting frequency and time, depending on their context. Therefore, we added an adjustable goal-setting element in our system.

Monitoring of participation was required or not depending on the individual. Thus, two options, one with and one without this element, were implemented as customizable in LiquidEye.

With regard to feedback, the kinds of feedback were not distinguishable. However, there was a difference between compliment feedback and others (descriptive message, comparative message, and evaluative message) according to most of the participants. Thus, we separated these two large categories in the setting options (Figure 1) and rotated the descriptive message, comparative message, and evaluative message.

There were opinions that there was no need to adjust the reward element depending on the context. If this element shows up in the system, it needs to keep showing up. All participants agreed that this element does not need to be customizable. Thus, it was kept as a basic setting.

The information delivery element (delivering health information) was required or not depending on the individual’s preference. Thus, two options, one with and one without this element, were implemented as customizable in LiquidEye.

For the notification window, the size of the window can influence acceptability. Preference regarding the notification element (popup and full screen) varied. Participants commented as follows:

If my previous work environment is paused by the system anyway, I rather prefer full-screen.

Participant #P1 and participant #P6

Interruption has to be as small as possible.

Participant #P7

On the settings page of the system (Figure 1), the following four options were provided: full-screen notification window, mid-size window, small message popup on the top right of the screen, and none.

Physical signals, such as blowing winds, were eliminated from our final list since they were rated below our borderline (score 4.0). In the end, only the sound element was implemented in LiquidEye. Users can select the sound option or not in the settings menu. Regarding message rotation, all participants insisted that it is a necessary function for all time points. Therefore, this was set as a basic function. For the aesthetic element (presence of the character, color, etc), preferences varied among the participants. For this reason, we made it customizable for users. Users can select the color of the screen and the kind of character they like or can eliminate it.

### Statistical Analysis With User Data Logs From Phase 2 (Real-World Deployment)

With LiquidEye, which was developed based on the focus group results, we collected user data logs during the 14 days of the experiment (n=680). To investigate interface elements that greatly affected the participation rate in the deployment, a multiple regression analysis was conducted with the users’ overall data logs. Each interface element (total 14 elements) was analyzed as an independent variable, and participation (1: participated, 0: did not participate) was analyzed as a dependent variable. Table 4 shows the results from the multiple regression analysis. We present results in line with our themes and subthemes defined above. The relevant elements included the instruction page of the eye resting strategy, goal setting for eye resting, compliment feedback after completing eye resting, mid-size popup window, and symptom-like visual effects that provide an alarm for the eye resting time.

**Table 4:** Results of multiple regression analysis.

Interface element (themes)	Interface element (subthemes)	Estimate	Standard deviation	*Z* value	*P* value
Intercept		−1.1114	0.6428	−1.73	.08
Education	Introduction page	0.6072	0.2469	2.46	.01
Goal setting	Default setting	−0.6647	0.2550	−2.61	.009
Goal setting	Adjusted setting	−0.0677	0.3730	−0.18	.86
Monitoring	Participation report	−0.1785	0.3697	−0.48	.63
Feedback	Default message	0.2460	0.2857	0.86	.39
Feedback	Compliment after eye resting	1.2977	0.3443	3.77	<.001
Information delivery	Health information	−0.6490	0.2824	−2.30	.02
Notification	Large-size window	−0.2572	0.3571	−0.72	.47
Notification	Mid-size window	−0.8873	0.3766	−2.36	.02
Notification	Small-size window	−0.6731	0.3798	−1.77	.08
Medium	Sound	0.2580	0.2297	1.12	.26
Aesthetic	Expert agent	−0.1277	0.3529	−0.36	.72
Aesthetic	Robot agent	−0.1270	0.3437	−0.37	.71
Aesthetic	Symptom-like effects with a notification window	0.7817	0.2714	2.88	.004

## Discussion

### Overview

Through two studies (ie, focus group study and deployment study), we explored the interface elements of computer-based interventions for CVS. Additionally, we collected real-world user data by deploying LiquidEye with customizable interface elements. With results from the deployment study, we could analyze how interface elements included in LiquidEye affected user participation with eye resting behavior. We will discuss the results while suggesting design guidelines for computer-based interventions for CVS.

### Guidelines for Important Interface Elements

A summary of design guidelines for interface elements is presented in Table 5.

Based on our results, we will discuss the effect of each interface element on user participation with LiquidEye. We will also share the user feedback from the 14-day experiment with LiquidEye to discuss the results. We will first discuss the interface elements that greatly affected participation in the eye resting behavior, including the instruction page of the eye resting strategy, goal setting for eye resting, compliment feedback after completing eye resting, mid-size popup window, and symptom-like visual effects that provide an alarm for the eye resting time.

Regarding the instruction page, most participants agreed that it helped a lot at the beginning of the experiment, but was no longer needed after participants got used to it. As participants mentioned, the adaptation level affects the consequences of the interface element *instruction page* by increasing user intervention coherence or increasing user burden. System designers should consider how fast users adapt to the system and, at the same time, how easy or hard the system has been designed since these factors influence a user’s need for the instruction page.

**Table 5:** Summary of design guidelines for interface elements.

Interface element (theme)	Example of interface element (subtheme)	Summary of design guidelines
Education	Introduction page	System designers should consider how fast users adapt to the system and, at the same time, how easy or hard the system was designed since these factors influence the user’s need for an instruction page.
Goal setting	Default setting, adjusted setting (customizable)	System designers should consider the user’s willingness to manage the eye condition since it decides a need for customization of goal settings. Default setting is the predefined setting regardless of the user’s autonomy.
Monitoring	Participation report	This element can be a double-edged sword for the motivation of the user. It can increase or decrease the self-efficacy of the user depending on the level of participation.
Feedback	Default message, compliment after eye resting	Depending on the context of the user, it can be either effective or ineffective. However, the preference for this element was high among users.
Information delivery	Health information	System designers should consider the user’s intention to manage the symptoms. If the user intention is high, the need for health information is also high at most times. However, low user intention can make users feel that this element is a burden.
Notification	Size of the window	The size of the popup influenced the forcefulness of the computer-based intervention. The full-screen notification with the high forcefulness was evaluated as most effective, but, at the same time, a high burden. Mid-size notifications positively affected user participation among other options.
Medium	Sound	The social context largely affected the user experience. Most of the participants insisted that it does not need to be in the system.
Aesthetic	Presence of characters (expert agent or robot agent) or visual effects (symptom-like effects)	Most of the time aesthetic elements rarely affect user participation, except when they strengthen the intervention effects by accompanying other intervention elements, such as the notification window in our case.

Goal setting for eye resting is another element that greatly affects user participation in eye resting behavior. It was interesting that the default setting for goal setting was relevant, while the adjusted setting was not. The default setting is a predefined setting based on the 20/20/20 rule (one should look at something 20 feet away for at least 20 seconds after 20 minutes of computer use) [30] for preventing or reducing CVS symptoms. Since the 20/20/20 rule is strict for long-time computer users as they have to rest three times per hour, we expected that customized settings (adjusted by users) would be more effective at increasing the participation rate. However, the customizable setting did not affect the user’s participation rate according to our statistical data. From user feedback, we found out that customizable goal setting (“adjusted” in Table 4) can result in increased effectiveness or increased burden depending on the attitude of the user. Users evaluated the system with the goal setting element more effectively when they were willing to manage their symptoms compared with those who were not willing to manage their symptoms. For example, participant P7 with a low attitude level showed a negative opinion. This participant made the following statement:

It is too annoying to set goals since I feel no need to manage my symptoms.

Compliment feedback after completing eye resting greatly affected user participation, but the qualitative results implied that it can sometimes be a burden for users. In particular, what users were doing right before the intervention affected the consequences of the feedback element. Participant A6 made the following statement:

I was working hard and then they told me to take a rest. I want to go back to my working environment as soon as the break is over. I don't feel like the extra things which are annoying and unnecessary.

However, most participants said that this element plays a positive role when they are not busy. Participant A5 made the following statement:

Compliment feedback was really helpful. I always turn this element on as my basic setting. It makes me feel good!

Regarding the interface element *notification window*, the mid-size popup window was related to user participation. The element was implemented in LiquidEye with four options (small-size popup, mid-size popup, full-screen popup, and no popup). Most of the participants insisted that the size of the popup influenced the forcefulness of LiquidEye. The full-screen popup with high forcefulness was evaluated as the most effective, but, at the same time, as having a high burden. Participant A10 made the following statement:

If I make up my mind to take a break anyway, I'd rather be forced to do it on time.

On the other hand, participant A6 made the following statement:

Small pop-up is barely noticeable, which makes me miss the participation.

Based on user feedback during and after the deployment study, we could infer that a mid-size popup window could be an alternative for the full-screen window and the small-size window with low effectiveness.

Another relevant interface element was symptom-like visual effects that provide an alarm for the eye resting time. An interesting opinion about this element was that its effectiveness depends on the size of the window and how often the effect is being rotated. Participant A9 made the following statement:

When it comes to this element, how much it grabs my attention matters. When it accompanies a full-screen popup window, it does not grab additional attention, because the popup window already fills my whole screen. However, when it accompanies small or middle size popup window, it strengthens the system to grab additional attention.

### Guidelines on Other Interface Elements

We are going to discuss additional findings on other interface elements even though they were not found to be relevant. They did not show significance, but monitoring the user’s participation and making a report on daily progress (“monitoring” in Table 4) can increase or decrease the self-efficacy of the user depending on the level of participation. Participant A3 made the following statement:

When I participated a lot, it was helpful for motivation but when I participated less, it was a burden to see.

Additionally, participant A2 made the following statement:

I just want it to show me the number of times I participated, not the rate of participation. It only gets lower if I do not participate in 100 percent.

The effectiveness of health information seems to depend on the intention to manage the symptoms. It could be effective if users are highly willing to manage their symptoms. Participant A11 made the following statement:

Getting this information makes me feel like I'm taking good care of my eyes. I spent more time thinking about my eyes.

However, for those who have a low intention of participating in eye resting behavior, health information could be a bothersome interface element. Participant A2 expressed the following negative opinion:

Whether it is health information or anything else, a lot of text could be the burden to use the system.

When it comes to a sound-based alarm (“sound” in Table 4), the social context largely affected the user experience. Most of the participants insisted that it does not need to be in the system. Participant A5 made the following statement:

I did not use it at least once since I always use my computer in my workplace.

Few participants mentioned that it can be assistive but it must be optional.

For the character-like agent (“expert agent” and “robot agent” in Table 4), there rarely existed comments from users. Participant A1 made the following comment:

It is barely noticeable. It does not affect my participation.

Additionally, users could customize their interfaces in the LiquidEye settings menu by themselves. This function of customization can increase effectiveness, but can be a burden depending on the clinical goal of the user. Most of the participants were satisfied with the customization options. Participant A4 made the following statement:

Depending on whether it is night or day, the desired setting is different since we are usually doing important things during the daytime and less important things during the nighttime.

On the other hand, participant A7 made the following statement:

It is a burden to change the options frequently. I want it to just recommend me the best option which is not very disturbing.

Avoiding work interruption was one of the major issues regarding user context. On the other hand, there is a need for a “right-on-time” intervention when it comes to clinical management of eye health. Participant A5 made the following statement:

I want it to show up right on time which is a most effective way for my eye health.

However, most participants agreed with the idea that LiquidEye needs to avoid critical moments (eg, sharing the monitor with colleagues in the middle of a conference). Participant A4 made the following statement:

Adding the do-not-disturb function to the LiquidEye will make the system more acceptable.

### Application to Other Clinical Symptoms

For developing our computer-based intervention, CVS was chosen as our condition of interest. Before expanding our results to other clinical domains that require computer-based interventions, designers or system developers should consider the below-mentioned steps.

First, when choosing interface elements for computer-based interventions, the initial thing to do is feature the clinical aim and the usage aim [29,32]. System designers need to decide on these aims and the intervention medium before they choose the interface elements. Depending on the clinical aim and target behavior, the intervention medium can be different, which means that a computer-based intervention is not the best medium for all cases.

Second, understanding the target clinical group is crucial [33]. Even if the same interface element is being used, implementation strategies have to differ depending on users’ unique features. Elements should be applied depending on the users’ personal and health behavior–related factors, such as attitude, behavior intention, and ultimate health goals. If the target user group is too heterogeneous, a computer-based intervention can be an option since it offers a great variety of options for assessing individuals, creating and delivering customized health messages, and providing individuals with the methods necessary to maintain or change their health-related behaviors [14].

Third, the evaluation of a computer-based intervention has to be completed before final implementation in a large population. Even when two computer-based interventions use the same framework, the consequences can be different. In our study, we evaluated the interface elements in LiquidEye with statistical analyses to better understand the consequences of choosing the interface elements.

If designers take all of the above points into consideration, our work is expected to decrease the cost of choosing interface elements in computer-based interventions by minimizing trial and error, even when implemented in other clinical domains.

### Conclusions

To reduce the prevalence of CVS in computer users, designing appropriate interventions that induce eye rest is one of the technology-based solutions. In this study, we suggested design implications to consider when designing a computer-based intervention for CVS. The sophisticated design of a customizable interface can make it possible for users to use the system more interactively, which can result in higher engagement. Among the various interface elements that are being implemented in computer-based interventions for CVS, we found that the instruction page of the eye resting strategy, goal setting for eye resting, compliment feedback after completing eye resting, mid-size popup window, and symptom-like visual effects that provide an alarm for the eye resting time greatly affected user participation in the eye resting behavior. We manually defined how these elements affected user participation based on the framework of acceptability. In a further study, we will explore the opportunities of automated technologies, such as facial expression recognition [34], deep sentiment analysis [35], and gaze-tracking algorithms [36], to detect positive or negative user experiences with the computer-based intervention. There are important technical challenges that still need to be addressed, but given the fact that this study was able to clarify the various factors related to computer-based interventions, the findings are expected to contribute greatly to the research of various computer-based intervention designs in the future.

## Abbreviations

CVS: computer vision syndrome
